# Maternal Lopinavir/Ritonavir Is Associated with Fewer Adverse Events in Infants than Nelfinavir or Atazanavir

**DOI:** 10.1155/2016/9848041

**Published:** 2016-04-04

**Authors:** Christiana Smith, Adriana Weinberg, Jeri E. Forster, Myron J. Levin, Jill Davies, Jennifer Pappas, Kay Kinzie, Emily Barr, Suzanne Paul, Elizabeth J. McFarland

**Affiliations:** ^1^Division of Infectious Diseases, Department of Pediatrics, University of Colorado School of Medicine and Children's Hospital Colorado, Aurora, CO 80045, USA; ^2^Department of Medicine, University of Colorado School of Medicine, Aurora, CO 80045, USA; ^3^Department of Pathology, University of Colorado School of Medicine, Aurora, CO 80045, USA; ^4^Department of Biostatistics and Informatics, Colorado School of Public Health, Aurora, CO 80045, USA; ^5^Department of Obstetrics and Gynecology, University of Colorado School of Medicine, Aurora, CO 80045, USA; ^6^Denver Health Medical Center, Denver, CO 80204, USA; ^7^Children's Hospital Colorado, Aurora, CO 80045, USA

## Abstract

Combination antiretroviral therapy (cART) is successfully used for prevention of perinatal HIV transmission. To investigate safety, we compared adverse events (AE) among infants exposed to different maternal cART regimens. We reviewed 158 HIV-uninfected infants born between 1997 and 2009, using logistic regression to model grade ≥1 AE and grade ≥3 AE as a function of maternal cART and confounding variables (preterm, C-section, illicit drug use, race, ethnicity, infant antiretrovirals, and maternal viremia). Frequently used cART regimens included zidovudine (63%), lamivudine (80%), ritonavir-boosted lopinavir (37%), nelfinavir (26%), and atazanavir (10%). At birth, anemia occurred in 13/140 infants (9%), neutropenia in 27/107 (25%), thrombocytopenia in 5/133 (4%), and liver enzyme elevation in 21/130 (16%). Corresponding rates of AE at 4 weeks were 59/141 (42%), 54/130 (42%), 3/137 (2%), and 3/104 (3%), respectively. Serious AE (grade ≥ 3) exceeded 2% only for neutropenia (13% at birth; 9% at 4 weeks). Compared with infants exposed to maternal lopinavir/ritonavir, infants exposed to nelfinavir and atazanavir had a 5-fold and 4-fold higher incidence of AE at birth, respectively. In conclusion, hematologic and hepatic AE were frequent, but rarely serious. In this predominantly protease inhibitor-treated population, lopinavir/ritonavir was associated with the lowest rate of infant AE.

## 1. Introduction

The prevention of perinatal transmission of HIV is one of the most successful public health interventions of the last few decades. The use of maternal combination antiretroviral therapy (cART), when combined with infant postnatal prophylaxis, has reduced transmission rates to less than 1% in developed countries [1, 2]. This is a remarkable achievement, but concerns remain regarding toxicity in these infants after exposure to multiple antiretrovirals (ARV) in utero, during delivery, and in early infancy [3].

Spontaneous preterm delivery and low birth weight have been associated with HIV infection during pregnancy. Although not a uniform finding across studies, the use of protease inhibitors (PI) during pregnancy may increase the incidence of preterm delivery [4–6]. Mitochondrial and nuclear genotoxicity are associated with in utero exposure to nucleoside reverse transcriptase inhibitors (NRTI) [7, 8]. In addition, laboratory adverse events (AE) have been described in ARV-exposed infants, including hematologic cytopenias and disruption of liver function. The NRTI are known to alter in vitro hematopoiesis [9, 10]. Transient macrocytic anemia was the most common side effect in infants exposed to zidovudine (ZDV) pre- and postnatally in the landmark PACTG 076 study [11, 12], and several reports describe profound neonatal anemia after ARV exposure in utero [13, 14]. Other studies have confirmed the link between ARV exposure and neonatal neutropenia, lymphopenia, and thrombocytopenia [15–20]. Liver dysfunction is described less frequently, although several studies have demonstrated elevated neonatal aspartate aminotransferase (AST), alanine aminotransferase (ALT), and bilirubin after exposure to perinatal ARV [21–25]. Increasing complexity of maternal cART correlates with an increased risk for hematologic and hepatic AE [15, 17, 21, 22, 26–28].

As new ARV and more complex cART regimens are administered for the prevention of perinatal HIV transmission, it is important to identify the toxicities associated with both established and novel regimens in order to inform best choices [29–31]. Prior studies have compared infant AE after exposure to different classes of ARV, for example, nonnucleoside reverse transcriptase inhibitor- (NNRTI-) based cART and PI-based cART [22, 27]. In this study, we examine infant AE after exposure to different maternal drugs within ARV classes.

## 2. Materials and Methods

### 2.1. Study Design

This study was approved by the Colorado Multiple Institutional Review Board and exempted from informed consent. This was a retrospective chart review of 190 pregnancies complicated by HIV infection that were managed by the Children's Hospital Immunodeficiency Program (CHIP) in Denver. CHIP is the reference center for the care of HIV-infected pregnant women in Colorado and neighboring states. Data were abstracted for all pregnancies from 1997 to 2009 that resulted in a live infant birth. Maternal data collected included demographics, ARV use, illicit drug use, mode of delivery, hematologic and hepatic laboratory values, CD4 count, and viral load. Undetectable viral load was defined as <400 copies/mL, as this was the lower limit of detection for the earliest data. Duration of maternal viremia during pregnancy was defined as the number of days from the estimated conception date until either the date of the first undetectable viral load measurement after which all subsequent viral load measurements were undetectable or, if there was no sustained viral suppression, the number of days between the estimated conception date and the date of infant birth. The estimated conception date was calculated from infant gestational age determined at birth. Infant data collected included gestational age (preterm defined as <37 weeks), birth weight (small for gestational age (SGA) defined as <3rd percentile of expected weight for gestational age), postnatal ARV prophylaxis, laboratory values (hemoglobin, neutrophil count, platelets, AST, ALT, and total bilirubin), and hospitalizations or illnesses. Infant complete blood count and liver function panel was assessed at birth (age 0–7 days) and at 4 weeks (±2 weeks). Laboratory toxicities were graded using the Division of AIDS Table for Grading the Severity of Adult and Pediatric Adverse Events [32]. If the medical record did not specify an upper limit of normal for AST and ALT, the limits imposed were 60 IU/L and 65 IU/L for AST and ALT, respectively, as these are the limits used by the Children's Hospital Colorado Laboratory. Infant infection status was monitored using HIV RNA and/or DNA PCR at birth, 2 weeks, 4 weeks, 6 weeks, and 4 months of age and HIV antibody testing starting at 12 months of age and repeated every 3–6 months until seroreversion was demonstrated.

### 2.2. Prophylaxis Regimens

Pregnant women received a clinician-prescribed antenatal ARV regimen that consisted of cART (≥3 ARV from ≥2 ARV classes) in most cases; modifications during the pregnancy were based on viral genotype, virologic response, safety, and tolerability, as previously described [33–35]. PI serum levels were routinely monitored and doses adjusted to achieve a trough above the 25th percentile for nonpregnant adults. Infant ARV exposure was assigned based on the maternal ARV received for ≥28 days during the 35 days immediately prior to delivery, thereby setting a minimum period of in utero drug exposure occurring near the time of infant laboratory assessment. Other maternal ARV received before the 35 days immediately prior to delivery was not accounted for in the analysis.

Most infants were prescribed postnatal prophylaxis consisting of six weeks of ZDV. In some infants, ZDV was replaced by stavudine (d4T) due to anemia, neutropenia, or both. In situations where the risk of perinatal transmission was increased, infants received two- or three-drug ARV. A detailed description of the infant postnatal prophylaxis prescribed and associated adverse events has been previously reported [36].

### 2.3. Statistical Analysis

The analysis included infants with at least one laboratory value at either birth or 4 weeks. The statistical analyses require that all observations (infants) are independent of one another or the results may be biased. Given that twins have identical exposure in utero and in order not to underestimate associations between AE and ARV, the twin with the lower grade toxicity was excluded from each pair. Maternal and infant characteristics were compared using *t*-tests, chi-square tests, and Fisher's exact tests, as appropriate. Because most infants were exposed to an antenatal ARV regimen containing two NRTI and a PI, AE were compared between infants exposed to different NRTI and between infants exposed to different PI. The number of infants exposed to nevirapine (*n* = 10) was too small for comparison. Rates of preterm birth and SGA were compared as a univariate analysis. Logistic regression was used to model grade ≥1 laboratory AE and grade ≥3 laboratory AE (yes/no) as a function of maternal ARV received for ≥28 days during the 35 days immediately prior to delivery and any confounding variable that changed the odds ratio (OR, 95% confidence interval) by >20%. Confounding variables used in the multivariate analyses include preterm birth, infant exposure to ZDV monoprophylaxis versus combination prophylaxis, mechanism of delivery, maternal race and ethnicity, maternal illicit drug use, and duration of maternal viremia during the pregnancy. Statistical significance was defined by *p* ≤ 0.05.

## 3. Results

### 3.1. Maternal Characteristics and Antiretroviral Regimens

One hundred and sixty-five mothers were managed at CHIP for 190 pregnancies, resulting in 196 live births. Of these, 163 infants (5 twins) had at least one laboratory value available for analysis. The analysis cohort consisted of 158 mother/infant pairs after excluding one infant from each twin pair. There were no perinatal transmissions of HIV. The infant demographics and maternal HIV and obstetrical characteristics are reported in Table 1. Severe maternal immune suppression was rare; only 7% of women had a CD4 count <200 cells/mm^3^ in early pregnancy. One hundred twenty-seven women (80%) received cART for at least 28 days prior to delivery. Fifteen women received cART for less than 28 days prior to delivery. Sixteen women received a less intensive ARV regimen. Maternal ARV received in the last month of pregnancy are detailed in Table 2. PI-based cART was received by 77% of women, including lopinavir with ritonavir (LPV/r, 37%), nelfinavir (NFV, 26%), atazanavir (ATV, 10%, including 2% without ritonavir), and saquinavir (4%). The majority of women received ZDV (63%), but 13%, 9.5%, and 4% received d4T, tenofovir (TDF), and abacavir (ABC), respectively. There were few differences in maternal and infant characteristics between the two largest treatment groups, LPV/r versus NFV (Supplementary Table  1 in the Supplementary Material available online at http://dx.doi.org/10.1155/2016/9848041).

### 3.2. Overall Frequency of Adverse Events

**Figure 1:**
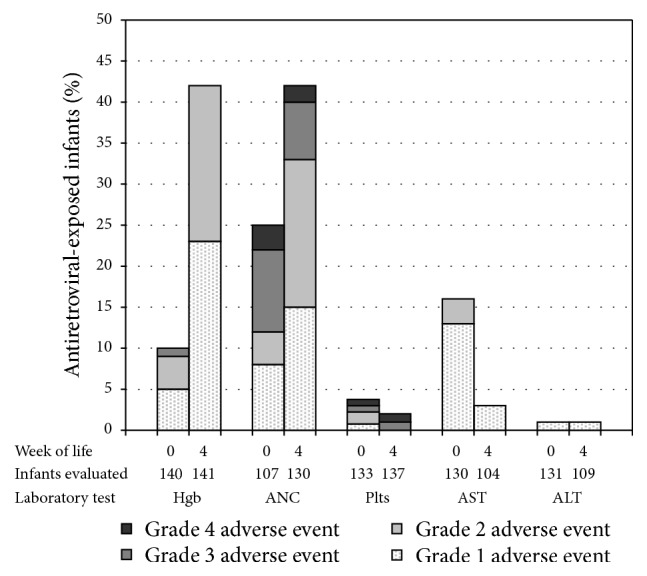
Frequency of infant adverse events. Bars represent percentages of infants with laboratory adverse events at birth (age 0–7 days) and at 4 weeks (±2 weeks). Hgb, hemoglobin; ANC, absolute neutrophil count; plts, platelet count; AST, aspartate aminotransferase; ALT, alanine aminotransferase.

Laboratory AE in the first four weeks of life were common, with three quarters of infants having an AE (any grade) at either birth or 4 weeks. The frequency of an AE grade ≥3 in any laboratory category was 12% at birth and 16% at 4 weeks. Neutropenia was most common, with 25% and 42% of infants having any grade AE, and 13% and 9% having grade ≥3 AE, at birth and 4 weeks, respectively (Figure 1). Anemia was the next most common, with 9% and 42% of infants having any grade AE, but only 1% and 0% having grade ≥3 AE, at birth and 4 weeks, respectively. Rates of AST AE fell from 16% at birth to 3% at 4 weeks. Rates of ALT AE and thrombocytopenia were low at birth and at 4 weeks. No infants had hyperbilirubinemia at birth.

The 26 (17%) infants born preterm were more likely than term infants to develop an AE of any grade (93% versus 71%, *p* = 0.03) or grade ≥3 (46% versus 17%, *p* = 0.003) at either birth or 4 weeks. Four preterm infants required blood transfusions due to severe anemia. No infants suffered a serious bacterial infection or a bleeding disorder as a complication of neutropenia or thrombocytopenia, and no infants developed liver failure as a result of hepatic inflammation. No infants developed renal or cardiac dysfunction.

### 3.3. Association of Maternal ARV with Infant Adverse Events

**Figure 2:**
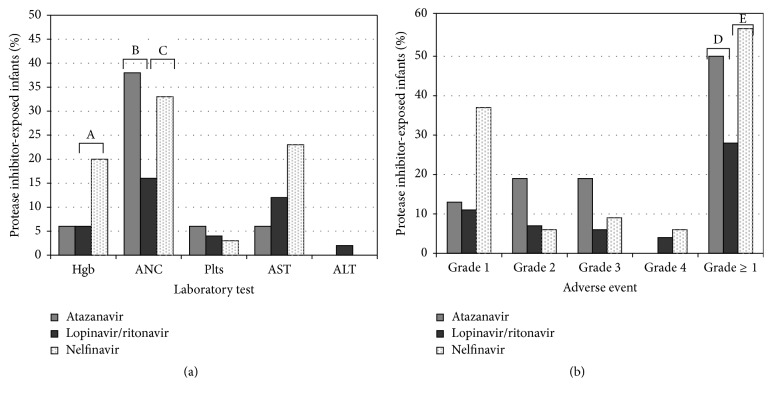
Frequency of infant adverse events at birth by maternal protease inhibitor. Bars represent the percentage of infants with a laboratory adverse event of any grade (a) or the percentage of infants whose most severe adverse event was represented by that particular grade (b). Number of infants per group: atazanavir ± ritonavir (16), lopinavir/ritonavir (54), and nelfinavir (35). Hgb, hemoglobin; ANC, absolute neutrophil count; plts, platelet count; AST, aspartate aminotransferase; ALT, alanine aminotransferase. ^A^OR = 7.4 (95% CI 1.4–39.0), *p* = 0.02; ^B^OR = 18.3 (95% CI 1.2–267.8), *p* = 0.03; ^C^OR = 10.6 (95% CI 1.7–66.4), *p* = 0.01; ^D^OR = 4.2 (95% CI 1.0–17.5), *p* = 0.046; ^E^OR = 5.3 (95% CI 1.9–14.9), *p* = 0.002.

Rates of infant laboratory AE relative to antenatal exposure to maternal ARV were compared in a multivariate analysis. At birth, infants exposed to maternal NFV compared to LPV/r had a higher rate of anemia (OR 7.4 (95% CI 1.4–39.0), *p* = 0.02), neutropenia (OR 10.6 (95% CI 1.7–66.4), *p* = 0.01), and any AE (OR 5.3 (95% CI 1.9–14.9), *p* = 0.002) (Figure 2). This association was maintained when limited to infants exposed to the same maternal NRTI backbone (ZDV and lamivudine) (Table 3). The difference was primarily the result of grade 1 AE; rates of grade ≥3 AE remained relatively low in both groups (9% versus 14% of LPV/r-exposed versus NFV-exposed infants, resp.; Figure 2). Exposure to maternal ATV was also associated with a higher rate of any laboratory AE compared to LPV/r (OR 4.2 (95% CI 1.0–17.5), *p* = 0.046) with the difference primarily due to higher rates of neutropenia (OR 18.3 (95% CI 1.2–267.8), *p* = 0.03) (Figure 2). At 4 weeks, there were no longer significant differences in AE between infants exposed to different PI (Table 4).

Additional maternal and infant characteristics were examined to determine whether variables not included in the multivariate analysis might contribute to differences in infant AE at birth for the NFV and LPV/r groups, which had a sample size large enough for analysis (Supplementary Table  1). The mean estimated duration of maternal viremia was longer in the NFV-exposed group than in the LPV/r-exposed group (172 versus 125 days, *p* = 0.05). However, the association of infant AE with NFV versus LPV/r exposure remained significant when duration of maternal viremia was included as a covariate in the multivariate analysis (OR 7.9 (95% CI 2.4–26.2), *p* = 0.0007). In addition, a univariate analysis demonstrated that the duration of maternal viremia did not correlate with the frequency of infant AE (OR 1.0 (95% CI 0.99–1.0), *p* = 0.57). An anticipated difference between the treatment groups was the year of delivery. NFV was prescribed in an earlier time period (1997–2007); LPV/r was first prescribed in 2003 and surpassed NFV as the most commonly used PI at our center by 2004. Other variables did not differ including the duration of antenatal exposure to NFV or LPV/r or the proportion of women who received the PI prior to conception (Supplementary Table  1).

Most women were treated with ZDV, but a substantial number received ABC, d4T, or TDF; therefore, rates of infant AE were compared between these groups. There were no significant differences at birth in rates of anemia, neutropenia, thrombocytopenia, or liver enzyme elevation between infants exposed to maternal ZDV compared with ABC, d4T, or TDF (Supplementary Table  2).

Rates of preterm birth and SGA for infants exposed to different maternal PI were compared in a univariate analysis. No significant differences were found in rates of preterm birth (5/16 (31%), 9/58 (16%), and 5/37 (14%), *p* = 0.26) or SGA (2/16 (13%), 7/56 (13%), and 4/35 (11%), *p* > 0.99) between infants exposed to ATV, LPV/r, and NFV, respectively, although the small sample size limited the power to detect a difference.

## 4. Discussion

Low-grade laboratory AE were common at birth and 4 weeks in the cART-exposed infants, but grade ≥3 AE were rare. Anemia and neutropenia made up the majority of AE, and neutropenia comprised nearly all grade ≥3 AE. Our results are in agreement with those of previous studies, which showed frequent, but low-grade, hematologic abnormalities among ARV-exposed infants. Pacheco et al. reported significantly decreased infant hemoglobin and neutrophil values, and Mussi-Pinhata et al. described anemia at hospital discharge in 24% of ARV-exposed infants, although in both studies nearly all AE were of grade 1 or 2 [17, 23]. Read et al. described grade ≥3 anemia or neutropenia in less than 10% of ARV-exposed infants in the first six weeks of life [19]. Four infants in our study required a blood transfusion due to anemia, all of whom were born at ≤32 weeks of gestation. Preterm infants are likely to require blood transfusion even when unexposed to ARV, with about 80% of US infants with birth weight <1500 grams requiring at least one transfusion [37]. None of the infants born ≥37 weeks of gestation in this study developed clinically significant AE. Although the majority of AE in these HIV- and ARV-exposed infants were of low grade, the mechanism underlying the AE is not fully understood and the long term effects of these early toxicities are unknown.

We found increased infant laboratory AE at birth associated with exposure to maternal NFV and ATV compared with LPV/r. The difference was observed even when the analysis was restricted to mother/infant pairs with the same NRTI background. At 4 weeks, no difference was detectable between these groups, suggesting that the effect is either transient or masked by the effect of postnatal ARV. Our multivariate analysis controlled for exposure to postnatal ZDV monoprophylaxis versus combination prophylaxis. The association of laboratory AE with particular antenatal ARV within the PI class is reported here for the first time. Of note, Bellon Cano et al. described increased anemia, thrombocytopenia, and liver enzyme elevation after exposure to PI-based cART (of which most regimens contained NFV) compared with two or three NRTI or NNRTI-based cART [22].

The mechanism of the PI effect on hematologic AE is unclear. Fetal hematopoiesis may be more susceptible to the effects of PI, as it occurs mainly in the liver and peripheral lymphoid tissues. PI cross the placenta to some degree; albeit due to their large molecular weight and high protein binding capacity, they do not reach therapeutic levels in cord blood. Cord blood to maternal plasma ratios for ATV, NFV, and LPV range between 0.13–0.24, 0.16–0.22, and 0–0.49, respectively [38]. Amniotic fluid to maternal plasma ratios have been described for NFV (0.14–0.44) and LPV (0.08) [39, 40]. Although serum levels for some PI may be low during pregnancy [41], in this cohort plasma PI levels were routinely monitored during pregnancy and doses adjusted to achieve a trough above the 25th percentile for nonpregnant adults. Therefore, the difference in infant AE is not likely attributable to differences in maternal PI drug levels.

Because this was a retrospective study, it is possible that the maternal receipt of various PI could be a marker for another confounding variable that contributed to infant AE. Examination for potential confounding variables between the NFV and LPV/r groups identified that on average women receiving NFV had a longer duration of viremia than women receiving LPV/r. However, viremia did not correlate with increased risk of infant AE, and inclusion of maternal viremia as a covariate did not alter the relationship of infant AE with exposure to NFV versus LPV/r.

Owing to its limited antiviral activity, NFV is no longer recommended for use in pregnant women [42]. However, there may be rare clinical scenarios in which NFV use might be considered, such as for patients who cannot tolerate or have resistance to other ARV that are included in the preferred and alternative regimens for pregnancy. The results of this study may be further rationale to avoid NFV during pregnancy.

We found that ATV exposure was associated with a higher incidence of neutropenia compared with LPV/r. However, infants in this study who were exposed to ATV did not show increased rates of liver enzyme elevation or hyperbilirubinemia compared with infants exposed to other PI. These findings are in contrast to those of Mandelbrot et al. who described a series of twenty-three infants exposed in utero to ATV, nine of whom developed elevated serum bilirubin concentrations at birth and five required phototherapy in the first three days of life [25]. Notably, a subsequent study found no association between ATV exposure and neonatal hyperbilirubinemia [43]. Our data need to be further confirmed in larger studies, because they were derived from only sixteen infants exposed to ATV, resulting in an OR with relatively large confidence intervals.

Although ZDV is commonly associated with hematologic side effects in exposed infants, we did not find more hematologic AE at birth among infants exposed to antenatal ZDV compared with those exposed to ABC, d4T, or TDF, but the number of infants exposed to the alternative NRTI was small. Few studies compare infant toxicities after in utero exposure to different NRTI, although Vivanti et al. demonstrated decreased genotoxicity in cord blood of infants exposed in utero to TDF versus ZDV [44].

Preterm birth has been described previously in association with in utero exposure to PI [4–6]. We did not find an association of preterm birth or SGA with LPV/r, NFV, or ATV, but the power to detect differences was limited by our small sample size. In addition, data describing other risk factors for preterm birth or SGA were not available in our data set. Perry et al. recently showed no differences in preterm birth or birth weight in infants exposed to maternal LPV/r versus ATV; this study did not report infant laboratory outcomes [45].

We acknowledge several limitations in this study. The sample size was relatively small. Mothers and infants were not randomized to various ARV in our study; they received a regimen that was clinically appropriate based on severity of disease, tolerability, viral response to therapy, and risk of perinatal HIV transmission. Although two groups of infants for whom we found a significant difference in AE (those exposed to LPV/r and NFV) were cared for in different time periods, we also found differences between the effect of LPV/r and ATV exposure, whose use was roughly contemporaneous. Despite these limitations, this study permits comparison of infant AE within classes of ARV, whereas most similar studies have compared between classes of ARV. It also provides data on newer ARV used for prevention of perinatal transmission.

## 5. Conclusions

In summary, when used in maternal cART for prevention of perinatal transmission of HIV, NFV and ATV were associated with increased infant AE compared to LPV/r. As ATV use in pregnant women is increasing, larger, preferably randomized studies of infants exposed to LPV/r versus ATV/r are needed to evaluate laboratory AE and to clarify any association with prematurity. Investigations into the safest maternal ARV regimens and timing of ARV initiation are important for optimizing outcomes for HIV-exposed infants, especially those with higher risk of hematologic and hepatic toxicity, such as preterm infants and infants in resource-limited settings.

## Supplementary Material

Supplementary Table 1 shows a comparison of maternal and infant characteristics for groups exposed to either lopinavir/ritonavir or nelfinavir.
Supplementary Table 2 shows the frequency of infant adverse events at birth associated with exposure to either maternal zidovudine or maternal abacavir, stavudine, or tenofovir.

## Figures and Tables

**Table 1:** Infant demographics and select obstetrical characteristics.

Characteristic	Number of infants/number evaluated^a^ (%)^b^
Race^c^	
Caucasian	92/158 (58%)
African American	53/158 (34%)
American Indian/Alaskan Native	2/158 (1%)
Other/unknown	11/158 (7%)
Ethnicity^c^	
Hispanic	55/158 (34%)
Not Hispanic	94/158 (60%)
Other/unknown	10/158 (6%)
Sex, male	81/152 (53%)
Entry maternal CD4 count^d^	
<200 cells/mm^3^	7/101 (7%)
200–500 cells/mm^3^	40/101 (40%)
>500 cells/mm^3^	54/101 (53%)
Entry median maternal HIV RNA copies/mL in plasma^d^ (range) *N* = 109	2120 (<20–213,191)
Antiretrovirals initiated before conception	44/152 (29%)
Maternal illicit drug use	19/158 (12%)
Mean gestational age in weeks (range) *N* = 150	37.7 (25–41.7)
Preterm deliveries	26/150 (17%)
Deliveries via Cesarean section	64/158 (41%)

^a^Denominator represents the number of infants with available data.

^b^Unless units of measurement are otherwise indicated.

^c^Infant race and ethnicity determined by maternal self-report.

^d^From earliest known maternal laboratory values during pregnancy.

**Table 2:** Antenatal antiretroviral exposure of infants.

Drug^a^	Number exposed (%) *N* = 158
Nucleoside reverse transcriptase inhibitors	
Abacavir	7 (4%)
Emtricitabine	15 (9.5%)
Lamivudine	126 (80%)
Stavudine	21 (13%)
Tenofovir	15 (9.5%)
Zidovudine	99 (63%)
Nonnucleoside reverse transcriptase inhibitors	
Nevirapine	10 (6%)
Protease inhibitors	
Atazanavir ± ritonavir^b^	16 (10%)
Lopinavir + ritonavir	59 (37%)
Nelfinavir	41 (26%)
Saquinavir ± ritonavir	7 (4%)

^a^Maternal treatment administered for ≥28 days of the 35 days immediately preceding delivery.

^b^Thirteen of sixteen women received ritonavir-boosted atazanavir.

**Table 3:** Infant adverse events at birth associated with maternal lopinavir/ritonavir versus nelfinavir, in combination with zidovudine and lamivudine^a^.

Laboratory test^b^	Lopinavir/ritonavir^c^	Nelfinavir^c^	Odds ratio (95% CI) *p* value
Hgb	2/34 (6%)	6/29 (21%)	6.3 (1.01–39.8) *p* = 0.049

ANC	2/22 (9%)	7/25 (28%)	N/A^d^ *p* = 0.14

AST	4/34 (12%)	7/25 (28%)	3.5 (0.73–16.7) *p* = 0.12

Highest grade AE, all tests	10/35 (29%)	17/29 (59%)	4.9 (1.6–15.4) *p* = 0.006

Hgb, hemoglobin; ANC, absolute neutrophil count; AST, aspartate aminotransferase.

^a^Multivariate analysis using logistic regression was used to model grade ≥1 AE (yes/no) as a function of maternal antiretroviral treatment. Groups restricted to infants born to mothers treated with zidovudine/lamivudine in combination with either lopinavir/ritonavir or nelfinavir.

^b^There were no adverse events for bilirubin, 1 adverse event for alanine aminotransferase in the lopinavir/ritonavir group, and 3 adverse events for platelet count in the nelfinavir group, not shown separately but included in the maximum adverse events.

^c^Number of infants with adverse event/number of infants exposed (%).

^d^Fisher's exact test reported.

**Table 4:** Infant adverse events at 4 weeks associated with exposure to maternal lopinavir/ritonavir versus nelfinavir or atazanavir^a^.

Laboratory test^b^	Lopinavir/ritonavir^c^	Nelfinavir^c^	Odds ratio (95% CI) *p* value^d^	Atazanavir^c^	Odds ratio (95% CI) *p* value^e^
Hgb	19/53 (36%)	14/34 (41%)	1.3 (0.52–3.0) *p* = 0.62	6/14 (43%)	1.3 (0.41–4.4) *p* = 0.63

ANC	24/48 (50%)	11/30 (37%)	0.58 (0.23–1.5) *p* = 0.25	5/14 (36%)	0.56 (0.16–1.9) *p* = 0.35

Bili	11/52 (21%)	9/26 (35%)	2.5 (0.75–8.1) *p* = 0.14	3/7 (43%)	2.0 (0.35–11.4) *p* = 0.44

Highest grade AE, all tests	38/55 (69%)	22/35 (63%)	0.76 (0.31–1.8) *p* = 0.54	9/14 (64%)	0.62 (0.16–2.4) *p* = 0.49

Hgb, hemoglobin; ANC, absolute neutrophil count; Bili, total bilirubin; AE, adverse event.

^a^Multivariate analysis using logistic regression was used to model grade ≥1 AE (yes/no) as a function of maternal antiretroviral treatment. No significant differences were found.

^b^There were no adverse events for alanine aminotransferase, 1 adverse event each for aspartate aminotransferase in the lopinavir/ritonavir and atazanavir groups, and 1 adverse event for platelet count in each group, not shown separately but included in the maximum adverse events.

^c^Number of adverse events/number exposed (%).

^d^Lopinavir/ritonavir versus nelfinavir.

^e^Lopinavir/ritonavir versus atazanavir.
