# Supplementary material for: Maternal Lopinavir/Ritonavir Is Associated with Fewer Adverse Events in Infants than Nelfinavir or Atazanavir
Source: Infect Dis Obstet Gynecol. 2016 Apr 4;2016:9848041. doi: 10.1155/2016/9848041 (PMC4834394; doi:10.1155/2016/9848041)
Supplement: Supplementary file 1 — Supplementary Table 1 shows a comparison of maternal and infant characteristics for groups exposed to either lopinavir/ritonavir or nelfinavir. Supplementary Table 2 shows the frequency of infant adverse events at birth associated with exposure to either maternal zidovudine or maternal abacavir, stavudine, or tenofovir. [file 9848041.f1.pdf]

Supplementary Table 1. Comparison of maternal and infant characteristics for lopinavir/ritonavir- and nelfinavir- exposed groups.

Characteristic	Number with characteristic/Number evaluated[a] (%)[b]		p-value
	Lopinavir/ritonavir	Nelfinavir	
Initial maternal CD4 count[c]			
Mean cells/mm^3 (range)	559 (15-1176)	496 (150-769)	0.27
CD4 <200 cells/mm^3	1/37 (3%)	1/22 (4%)	
CD4 200-500 cells/mm^3	14/37 (38%)	7/22 (32%)	0.90
CD4 >500 cells/mm^3	22/37 (59%)	14/22 (64%)	
Mean days of detectable maternal viremia during pregnancy[d] (range)	125 (0-274)	172 (0-288)	**0.05**
Maternal viral load detectable at any time during pregnancy[d]	29/51 (57%)	24/31 (77%)	0.09
Entry maternal viral load detectable[c,d]	25/50 (50%)	15/23 (65%)	0.11
Maternal viral load detectable ≥90 days after antiretroviral initiation[d]	5/41 (12%)	6/21 (29%)	0.16
Maternal viral load detectable within 28 days of delivery[d]	3/32 (9%)	2/18 (11%)	>0.99
Mean days of antenatal antiretroviral therapy during pregnancy (range)	170 (40-287)	167 (46-287)	0.86

Mean days of lopinavir/ritonavir or nelfinavir during pregnancy (range)	152 (29-280)	156 (28-287)	0.81
Lopinavir/ritonavir or nelfinavir initiated prior to conception	16/54 (30%)	11/36 (31%)	>0.99
Maternal co-infection with Hepatitis B	1/45 (2%)	1/21 (5%)	0.54
Maternal co-infection with Hepatitis C	4/41 (10%)	2/27 (7%)	>0.99
Mean maternal age at delivery, years (range)	29.6 (20.2-40.6)	30.7 (21.4-43.3)	0.37
Maternal parity, mean number of prior deliveries (range)	1.6 (0-5)	1.6 (0-4)	0.91
Male infants (%)	24/51 (47%)	15/34 (44%)	0.83
Infant birthweight			
Small for gestational age	7/52 (13%)	3/29 (10%)	
Appropriate for gestational age	45/52 (87%)	25/29 (86%)	0.46
Large for gestational age	0/52 (0%)	1/29 (3%)	

[a]Denominator represents the number of infants with available data.

[b]Unless units of measurement are otherwise indicated.

[c]Earliest known maternal laboratory values during pregnancy.

[d]Detectable viral load defined as ≥400 copies/mL, as this was the limit of detection for the earliest data.

Supplementary Table 2. Frequency of infant laboratory adverse events at birth associated with exposure to maternal zidovudine vs. abacavir, stavudine, or tenofovir.

Laboratory test[a]	Zidovudine[b]	Abacavir, stavudine, or tenofovir[b]	Odds Ratio (95% CI) p-value
Hgb	10/85 (12%)	1/35 (3%)	4.7 (0.58-37.9) p=0.15
ANC	10/67 (15%)	10/26 (38%)	0.36 (0.10-1.3) p=0.11
Plts	3/80 (4%)	1/35 (3%)	1.3 (0.13-13.2) p=0.81
AST	14/76 (18%)	2/35 (6%)	3.7 (0.80-17.4) p=0.09
Highest grade AE, all tests	32/86 (37%)	12/36 (33%)	2.1 (0.78-5.4) p=0.14

Multivariate analysis using logistic regression was used to model grade ≥1 AE (yes/no) as a function of maternal antiretroviral treatment. No significant differences were found.

Hgb, hemoglobin; ANC, absolute neutrophil count; Plts, platelets; AST, aspartate aminotransferase; AE, adverse event.

[a]There was 1 adverse event for alanine aminotransferase in the ZDV group, not shown separately but included in the maximum adverse events.

[b]Number of adverse events / Number exposed (%).
